# Maxillofacial Traumatic Fractures in a Saudi Pediatric Subpopulation: A 10-Year Retrospective Study

**DOI:** 10.7759/cureus.46002

**Published:** 2023-09-26

**Authors:** Abdulazez A Aleid, May W Al-Khudhairy, Haitham Bin Turaiky, Muslat A Bin Rubaia’an

**Affiliations:** 1 Oral and Maxillofacial Surgery and Diagnostic Sciences, Riyadh Elm University, Riyadh, SAU; 2 Oral and Maxillofacial Surgery, King Fahad Specialist Hospital, Tabuk, SAU; 3 Oral and Maxillofacial Surgery, King Saud Medical City, Riyadh, SAU; 4 College of Medicine and Dentistry, Riyadh Elm University, Riyadh, SAU

**Keywords:** epidemiology, analysis, face, children, trauma

## Abstract

Aim: The primary objective of this study was to analyze the patterns and etiology of maxillofacial fractures among hospitalized pediatric patients.

Methods: A retrospective analysis was conducted on pediatric trauma cases admitted to King Saud Medical City, Riyadh, Kingdom of Saudi Arabia, from January 1, 2010 to December 31, 2020.

Data extracted from medical records was utilized to identify and establish correlations with various variables, including age, gender, trauma etiology, and the type of sustained fracture.

Results: The study involved a total of 167 patients. The mean age of the patients was 11.79 years, with the youngest and oldest patients being 2 and 17 years old, respectively. The majority of participants (70.7%, n = 118) were male. A total of 257 fractures were identified, with the highest number (n = 173, 67.3%) occurring in the lower third of the face.

Conclusion: Road traffic accidents (RTAs) emerged as the primary cause of trauma in our study, accounting for 73% (n = 122) of cases, followed by falls at 16.8% (n = 28). All instances of polytrauma were linked to RTAs. Fractures in the lower third of the face constituted over two-thirds (67.3%, n = 173) of the total fractures observed. Among the fractures, condyle fractures were the most frequently observed (19.8%, n = 51) in our study.

## Introduction

Maxillofacial fractures often lead to high morbidity, functional impairment, physical deformity, and psychological trauma [1,2]. Oral and maxillofacial injuries encompass harm inflicted upon the mouth’s soft tissues, teeth, the facial skeletal structure, and related soft tissues within the head and neck region [3]. The distinctiveness of maxillofacial injuries arises from the intricate anatomy of this region [4]. It is estimated that between 1% and 15% of facial fractures occur in pediatric populations [5]. The reduced frequency of maxillofacial injuries in pediatric populations has been attributed to several factors, such as a higher cranium-to-face ratio, skeletal flexibility, and having more parental and school care [6,7]. Diagnosing and managing facial fractures in pediatric patients introduces challenges distinct from those encountered in the adult population. This context necessitates careful attention to several pivotal factors, encompassing anatomical, physiological, and psychological aspects of growth and the intricacies inherent in trauma [8,9].

The presence of underdeveloped craniofacial features and variations in skeletal physiology significantly influence the occurrence, severity, and site of facial injuries among the pediatric population. Mid-facial fractures are relatively infrequent in pediatric cases due to the protective role played by the mandible and cranium, which absorb most of the impact from traumatic incidents. Additionally, these mid-facial bones possess remarkable elasticity. In contrast, mandibular fractures constitute the most prevalent type of facial fracture due to their bony prominence, necessitating hospitalization among pediatric patients [10]. Accurately identifying the anatomical location of mandibular fractures holds immense importance, as undiagnosed or inadequately treated mandibular trauma could potentially contribute to the development of progressive growth abnormalities in the facial region. Mandible fractures in young children typically manifest as “greenstick” fractures and tend to be solitary rather than the multiple-site fractures that are often seen in adults [11].

Numerous studies have consistently indicated that a significant proportion of maxillofacial trauma is linked to road traffic accidents (RTAs), irrespective of age or gender [12-15]. Moreover, the literature highlights other noteworthy contributors to maxillofacial trauma, such as injuries resulting from falls, sports-related incidents, assaults, instances of abuse, and animal bites [13].

Geographical variations in the occurrence and attributes of maxillofacial trauma can be ascribed to diverse socioeconomic, cultural, and environmental factors [6]. Gathering epidemiological data on maxillofacial injuries is pivotal to improving patient care and devising preventive measures [6]. However, a notable dearth of research exists when exploring the epidemiology of maxillofacial trauma in pediatric populations within Riyadh City, Saudi Arabia.

Uncovering a disease’s etiological and epidemiological aspects within a particular geographical area yields vital insights essential for devising suitable prevention and diagnostic approaches [16]. Consequently, this study aimed to examine the prevalence, frequency, and patterns of maxillofacial fractures within a pediatric subpopulation in Saudi Arabia. Furthermore, the study aimed to explore the correlation between various types of fractures and the underlying causes of trauma.

## Materials and methods

Ethical approvals

The study (FPGRP/2021/634/625/604) received approval from the Institutional Review Board (IRB) of Riyadh Elm University. Furthermore, the study also gained approval from the research center committee at King Saud Medical City (KSMC), and it was duly registered under IRB Number H-01-R-053.

Study setting

The research was carried out at KSMC, where all patients aged 17 and under who had been admitted for having maxillofacial trauma were included in the study. This age group aligns with the classification of the pediatric population as outlined in the Convention on the Rights of the Child, as adopted by the United Nations General Assembly [17]. These patients underwent open or closed reduction procedures in the operating rooms of KSMC, located in Riyadh, Kingdom of Saudi Arabia (KSA), between January 1, 2010 and December 31, 2020. The retrieved records were carefully reviewed and analyzed. To confirm the diagnoses, computed tomography scans were scrutinized. Essential data about each participant, including age, gender, injury etiology, and the nature of maxillofacial fractures, were extracted from the medical records. The injury etiologies were categorized into several groups: RTA, fall, assault, abuse, sports-related injuries, and animal attacks. Additionally, the observed patterns of maxillofacial fractures were broadly classified into upper, middle, and lower thirds of the face fractures.

Exclusion criteria

The following cases were not considered in this study: patients with a pre-established history of skeletal diseases that led to abnormal or unnatural bone growth, such as Osteogenesis Imperfecta; patients with pathologic fractures, including fractures occurring in areas affected by osteomyelitis, were excluded from the study; patients who had isolated dentoalveolar fractures, such as dental trauma, were also excluded (these cases were managed in outpatient settings or the Emergency Room [ER]); patients with isolated nasal bone fractures were excluded, as such cases fall under the jurisdiction of the Otorhinolaryngology department at KSMC; and patients with isolated soft tissue injuries, such as lacerations, were not included in the study, given that they received treatment in the ER. Additionally, patients without artifact-free computed tomography records were excluded.

Study variables

The following information was collected: age in years, type of injury (categorized as hard tissue with no soft tissue injury requiring intervention or soft and hard tissue), etiology of injury (including RTA, fall trauma, assault, abuse, sports injuries, and animal attacks), trauma specificity (differentiated between isolated maxillofacial trauma, confined to the facial skeleton and related soft tissue, and polytrauma, involving two or more body regions), and anatomic location of fractures.

The anatomic location of fractures included the following classifications: frontal bone fractures (subdivided into the anterior wall of the frontal sinus, posterior wall of the sinus, and involvement of both walls) [18], maxillary fractures (further classified based on René Le Fort’s system as type I, II, and III) [19], zygomaticomaxillary complex (ZMC) fractures (categorized as low-energy, middle-energy, and high-energy fractures) [20], nasoorbitoethmoid (NOE) (additionally categorized as type I, II, and III) [21], orbital fractures (grouped as linear, blow-out, and complex fractures) [5,22], fractures of the mandible (classified according to the Ivy and Curtis classification, including symphysis, parasymphysis, body, angle, ramus, neck of the condyle, and coronoid process) [5,23].

Statistical analysis

The authors centralized the extracted patient data digitally using Microsoft Excel software. Subsequently, data analysis was conducted using IBM Corp. Released 2015. IBM SPSS Statistics for Windows, Version 23.0. Armonk, NY: IBM Corp. Descriptive statistics were computed, including the mean, standard deviation, frequency, and percentage. The Pearson chi-square test was utilized to compare categorical data across two or more groups. An unpaired t-test was applied to compare means between two groups, while the analysis of variance (ANOVA) test was employed for comparing means across more than two groups. A p-value of less than 0.05 determined significance.

## Results

During the study period, 233 patients younger than 17 years were initially screened by a maxillofacial surgeon. Sixty-six patients were excluded because they met the exclusion criteria. Hence, a total of 167 patients’ records were analyzed for the present study. Among these were 118 males (70.7%) and 49 females (29.3%). The sample exhibited a higher representation of males, resulting in an overall male-to-female ratio of 2.4:1. The average age of the patients was 12 years, while the youngest and oldest patients were 2 and 17 years old, respectively (Table 1).

**Table 1:** Descriptive statistics of age

Age	Mean		11.790
	95% confidence interval for mean	Lower bound	11.179
		Upper bound	12.401
	Median		13
	Standard deviation		3.999
	Minimum		2
	Maximum		17

Most of the sample had solely hard tissue injuries (85.6%, n = 143), whereas only 14.4% (n = 24) experienced soft and hard tissue injuries. RTAs accounted for the majority of trauma cases (73%, n = 122), with falls comprising the second most common cause (16.8%, n = 28). Notably, no abuse, sports-related injuries, or animal attacks were documented. Trauma etiologies had no strict patterns over the whole study period. In addition, 41.9% (n = 70) of the patients exhibited isolated facial trauma, while the remaining 58.1% (n = 97) presented with polytrauma (Table 2).

**Table 2:** Descriptive characteristics of study participants. Data are presented as frequencies (n) and percentages (%). RTA: Road traffic accident.

Descriptive characteristics		N	%
Type of injury	Hard tissue	143	85.6%
	Soft and hard tissue	24	14.4%
Etiology	Assault	17	10.2%
	Fall	28	16.8%
	RTA	122	73%
	Animal bites	0	0%
	Abuse	0	0%
	Sports injuries	0	0%
Trauma specificity	Isolated facial trauma	70	41.9%
	Polytrauma	97	58.1%

Among the male patients, the prevalence of exclusive hard tissue injuries (88.1%, n = 104) surpassed that of combined soft and hard tissue injuries (11.9%, n = 14). A similar pattern was noted among female patients, where the proportion of exclusive hard tissue injuries stood at 79.6% (n = 39), while the combined soft and hard tissue injuries accounted for 20.4% (n = 10). The disparities in these injury types did not exhibit statistical significance (p=0.152). Notably, the incidence of assaults was higher among males (13.6%, n = 16), whereas falls were more common among females (26.5%, n = 13). In contrast, the prevalence of RTAs remained nearly identical between both genders, revealing statistical significance (p=0.014). Furthermore, the occurrence of polytrauma was slightly elevated among male participants (60.2%, n = 71) compared to females (53.1%, n = 26), yet this distinction lacked statistical significance (p=0.397) (Table 3).

**Table 3:** Distribution of study variables according to gender Data are presented as frequencies (n) and percentages (%). Chi-square test, *Statistically significant at p≤0.05 RTA: Road traffic accident.

Study Variables		Gender				P-value
		Female		Male		
		N	%	N	%	
Type of injury	Hard tissue	39	79.6%	104	88.1%	0.152
	Soft and hard tissue	10	20.4%	14	11.9%	
Etiology	Assault	1	2.0%	16	13.6%	0.014*
	Fall	13	26.5%	15	12.7%	
	RTA	35	71.4%	87	73.7%	
Trauma specificity	Isolated facial trauma	23	46.9%	47	39.8%	0.397
	Polytrauma	26	53.1%	71	60.2%	

Over 95% (n = 23) of cases involving a combination of soft and hard tissue injuries, as well as approximately 69% (n = 99) of instances of exclusive hard tissue injuries, could be attributed to RTAs. Falls were responsible for nearly 19% (n = 27) of hard tissue injuries, and only 4% (n = 1) of cases involving both soft and hard tissue injuries were linked to falls. This variance in etiological distribution held statistical significance (p=0.024). Notably, among patients with soft and hard tissue injuries, 87.5% (n = 21) exhibited polytrauma, while for those with exclusive hard tissue injuries, the figure was 53.1% (n = 76). This discrepancy also showed statistical significance (p=0.002). Detailed data is provided in Table 4.

**Table 4:** Association of study variables, etiology, and trauma specificity with type of injury Data are presented as frequencies (n) and percentages (%). Chi-square test, *Statistically significant at p≤0.05 RTA: Road traffic accident.

Study Variables		Type of injury				P-value
		Hard tissue		Soft & Hard tissue		
		N	%	N	%	
Etiology	Assault	17	11.9%	0	0%	0.024*
	Fall	27	18.9%	1	4.2%	
	RTA	99	69.2%	23	95.8%	
Trauma specificity	Isolated facial trauma	67	46.9%	3	12.5%	0.002*
	Polytrauma	76	53.1%	21	87.5%	

Every instance of polytrauma (100%, n = 97) stemmed from RTAs, whereas only 35.7% (n = 25) of isolated facial trauma cases were attributed to the same etiology. Notably, most isolated facial trauma cases resulted from falls (40%, n = 28). This variation proved statistically significant (p=0.001) (Table 5).

**Table 5:** Association between etiology and trauma etiology Data are presented as frequencies (n) and percentages (%). Chi-square test, *Statistically significant at p≤0.05 RTA: Road traffic accident.

Etiology	Trauma Specificity				P-value
	Isolated facial trauma		Polytrauma		
	N	%	N	%	
Assault	17	24.3%	0	0.0%	0.001*
Fall	28	40.0%	0	0.0%	
RTA	25	35.7%	97	100.0%	

**Figure 1:**
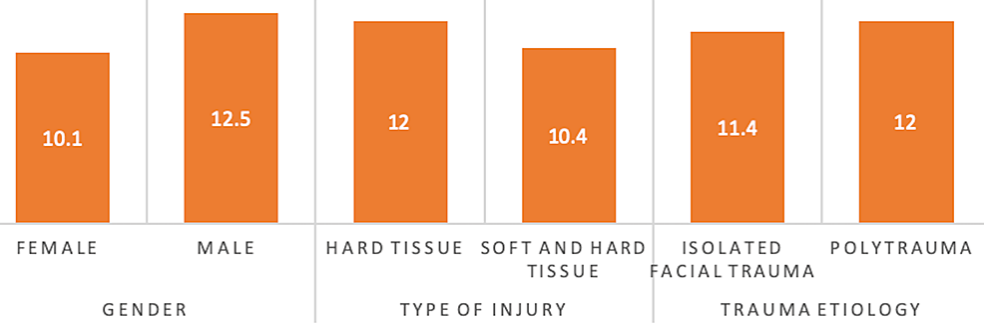
The age difference between categories of study variables Data are presented as mean.

The average age of males was 12.5 years, slightly surpassing that of females at 10.1 years. Notably, patients who encountered soft and hard tissue injuries had a younger mean age of 10.4 years, unlike patients with exclusive hard tissue injuries, who exhibited an average age of 12.0 years (Figure 1).

A collective count of 257 (100%) fractures was identified. Notably, no middle-face fractures were attributed to falls, while fractures in the upper third of the face exclusively resulted from RTAs. Within the middle third of the face, middle-energy ZMC fractures constituted 26 cases (10.1%). Conversely, the upper third of the face exhibited the lowest incidence, with only 10 cases (3.8%). In contrast, the lower third of the face accounted for the majority, with 173 fractures (67.3%). The lower third of the face demonstrated the highest frequency of fractures across all causal factors. The condyle emerged as the most frequently fractured site, with 51 instances (19.8%) (Table 6).

**Table 6:** Distribution of fractures per etiology Data are presented as frequencies (n) and percentages (%). RTA: Road traffic accident; ZMC: Zygomaticomaxillary complex; NOE: Nasorbitalethmoid.

Site of fracture	RTA	Fall	Assault	Total	
Upper third of the face					
Anterior wall of frontal sinus	2	0	0	2	0.778%
Posterior Wall of frontal sinus	0	0	0	0	0%
Anterior and posterior walls	8	0	0	8	3.112%
Total	10	0	0	10	3.891%
Middle third of the face					
Le fort I	4	0	0	4	1.556%
Le fort II	6	0	0	6	2.334%
Le fort III	5	0	0	5	1.945%
Low energy ZMC	3	0	0	3	1.167%
Middle Energy ZMC	23	0	3	26	10.116%
High energy ZMC	10	0	1	11	4.280%
Type I NOE	0	0	0	0	0%
Type II NOE	1	0	0	1	0.389%
Type III NOE	0	0	0	0	0%
Linear Orbital	4	0	1	5	1.945%
Blow-out Orbital	7	0	5	12	4.669%
Complex Orbital	1	0	0	1	0.389%
Total	64	0	10	74	28.793%
Lower third of the face					
Symphysis	7	8	0	15	5.836%
Parasymphysis	32	6	2	40	15.564%
Body	33	4	4	41	15.953%
Angle	20	0	3	23	8.949%
Ramus	2	1	0	3	1.167%
Condyle	27	22	2	51	19.844%
Coronoid	0	0	0	0	0%
Total	121	41	11	173	67.315%

The variation in age across different anatomical fracture locations displayed statistical non-significance (p=0.068). Patients with zygomaticomaxillary complex (ZMC) fractures registered the highest mean age, trailed by those with multiple and frontal bone fractures (Table 7).

**Table 7:** Association between anatomic location of fractures and age Data are presented as the mean and standard deviation. ZMC: Zygomaticomaxillary complex; NOE: Nasorbitalethmoid; SD: Standard deviation. ANOVA test, *Statistical significance p≤ 0.05

Anatomic Location of Fracture	Age		P-value
	Mean	SD	
Frontal	12.5	5.1	0.068
Maxilla	9.4	5.7	
ZMC	13.9	2.4	
NOE	12.0	0	
Orbital	11.2	3.4	
Mandible	11.2	4.0	
Multiple fractures in the face	12.8	4.0	

## Discussion

Humans undergo continuous growth, leading to noticeable anatomical and structural variations that necessitate special consideration during the diagnosis and design of medical treatments (Figure 1).

**Figure 2:**
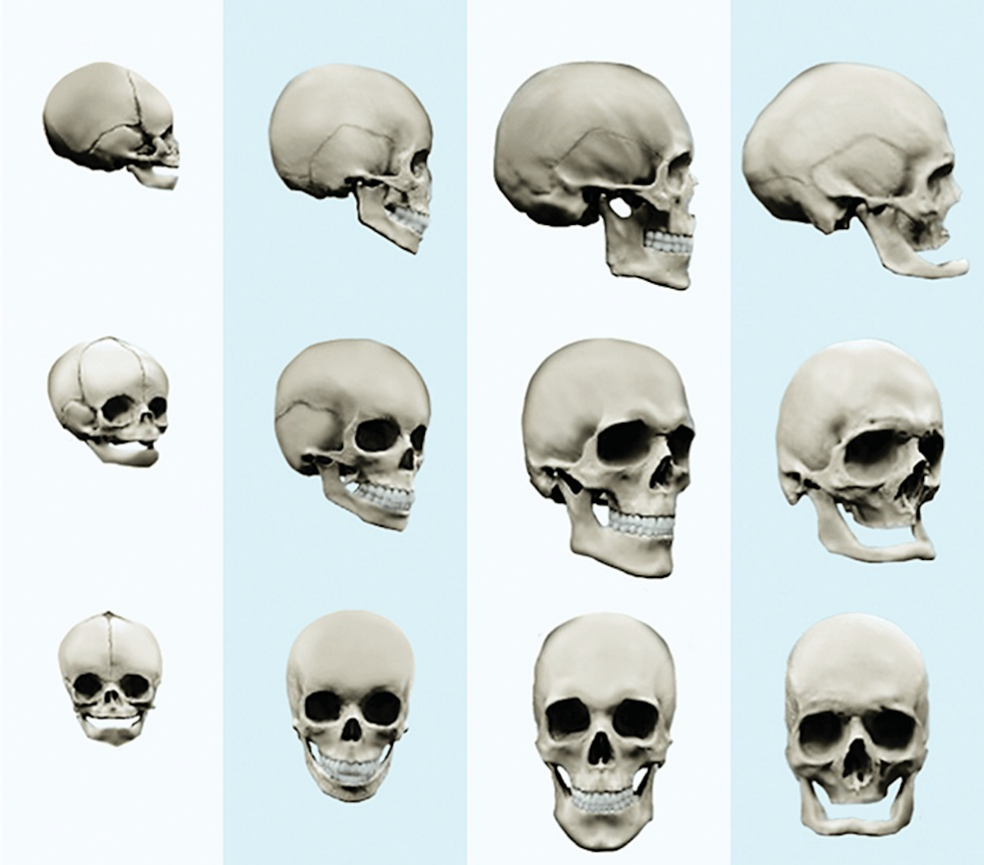
Age-related changes in human skull morphology This figure was reproduced from dos Santos Silva et al. [24] with the permission of John Wiley & Sons, Inc. (License Number: 5620760031569)

This study comprehensively analyzes the prevalence, frequency, and patterns of maxillofacial fractures in a pediatric subpopulation at KSMC in Riyadh, KSA. KSMC serves as a tertiary center and is one of KSA’s busiest medical facilities. Being a significant trauma center, it was chosen as the study site due to the high volume of cases it handles daily.

In the realm of children, trauma is associated with significant mortality and morbidity. Although maxillofacial injuries are rare among pediatric patients, their presence can lead to functional impairments and aesthetically unfavorable results. Previous studies have been conducted to shed light on the prevalence and characteristics of maxillofacial injuries within the pediatric population, including the pediatric population of Saudi Arabia [6,9,12,15,25-28].

This study aimed to retrospectively evaluate maxillofacial injuries in a sample of pediatric patients, specifically those under 17 who had been admitted to KSMC for having maxillofacial trauma. The average age of the individuals within the selected sample was determined to be 11.79±3.99 years. This finding is comparable to a previous study investigating a cohort of children aged 0 to 18 years, which reported a mean age of 13 years [29]. Additionally, it is relatively comparable to the findings of Daniels et al., who examined a sample from southern Saudi Arabia, aged 0 to 19 years, and reported a mean age of 14.4 years [15]. The elevated prevalence of fractures within this age group can be linked to alterations in lifestyle patterns stemming from increased engagement in social activities in addition to a general decline in parental oversight [8].

While epidemiology is subject to multiple variables, our study underscores the significant role that epidemiological factors play in distinguishing the outcomes of maxillofacial trauma. The current study revealed a notable male predominance of over 70% (n = 118) in the incidence of maxillofacial trauma, in line with previous findings [30]. Our research indicated a male-to-female ratio of 2.4:1. This ratio is comparatively lower than ratios previously reported in western Saudi Arabian cities like Jeddah (4:1) and Al-Madina (4.8:1) [15,27,28]. Interestingly, studies conducted in southern Saudi Arabian cities indicated much higher male-to-female ratios, such as Aseer (10:1) and Najran (16.6:1) [15,26]. This discrepancy could be attributed to the greater cultural diversity and outdoor activities for females in Riyadh and western cities compared to the more conservative populations of southern cities [15].

Moreover, the global prevalence of maxillofacial bone fractures tends to be higher in males than in females [15]. Several factors contribute to the observed male predominance. It has been posited that females often experience earlier maturation than males [9]. Additionally, historical, cultural, and social norms have restricted women’s participation in outdoor activities without supervision [9].

RTAs stand as the primary cause of child mortality, a fact highlighted by the World Health Organization (WHO) through data sourced from the National Center for Health Statistics in the United States of America [31]. Within the scope of this study, RTA accounted for a significant portion of maxillofacial trauma cases (73.1%, n = 122). This finding receives support from earlier investigations into the etiology of pediatric maxillofacial trauma [14,15,32]. The most recent census conducted by the Ministry of Health in the KSA as of May 2021 revealed the occurrence of 4,230, 4,549, and 3,202 RTA-related incidents involving individuals under 18 years of age in the years 2018, 2019, and 2020, respectively. Among these, 816, 827, and 587 cases led to fatalities [33]. Among the 257 fractures studied, 195 (75.8%) were attributed to RTAs. This underscores the critical need for preparation in situations where a child is involved in an RTA without the protection of a child’s seat or seatbelt. This issue is further exacerbated in Riyadh City due to the scarcity of pedestrian bridges and crossings. The potential consequences of a child arriving at an emergency department with multiple facial fractures can be disastrous for the child and his or her family. RTAs are influenced by many factors, ranging from driver negligence to unstable traffic regulations and the surge in daily vehicular operations, all contributing to the elevated frequency of such incidents. Community awareness campaigns that stress to parents and caregivers the significance of utilizing various self-protection measures may, however, prevent these incidents [34].

Falls emerged as the second most prevalent cause of trauma in this study (16.8%, n = 28), aligning with the findings of AlAli et al. but diverging from results reported by Wymann et al. and Mukhopadhyay et al., whose studies identified falls as the primary cause of pediatric maxillofacial trauma [6,32,35]. Notably, our study’s mean age of individuals affected by falls was 9.3 years, highlighting that fall-related trauma is more prominent in the younger pediatric age group due to their active lifestyle and adventurous nature. In addition, our study revealed that fall-induced trauma predominantly resulted in isolated facial injuries without accompanying injuries elsewhere in the body.

Assault was the least prevalent factor in our study, accounting for only 17 reported cases (10.2%), with no documented instances of adult-inflicted assault. Notably, assault-related maxillofacial trauma is closely linked with alcohol consumption [1]. The well-established connection between alcohol and interpersonal violence has been demonstrated by research from various nations, which underscores the increasing correlation between alcohol usage and maxillofacial trauma [1,36]. While Saudi Arabia maintains stringent laws against alcohol consumption, and although instances of interpersonal violence do exist, comprehensive research in this area remains relatively limited. Within our research, no instances were recorded as abuse. Whether this scarcity of reporting stems from the societal stigma surrounding such cases or from the infrequent nature of such incidents in Saudi Arabia is a subject that warrants further investigation through more extensive and multi-center studies. Examining the study conducted by Barbi et al., their focus centered on 250 children aged 5 to 16 who were victims of abuse or neglect, revealing that 72 of these children presented with lacerations and 18 with dento-alveolar fractures [37]. Child abuse encompasses emotional, physical, economic, and sexual mistreatment of individuals below 18 years of age, constituting a global epidemic [38]. A noteworthy majority of reported cases involving child abuse exhibit craniofacial trauma, leading to tangible physical consequences [39]. For a skilled dentist, recognizing orofacial injuries presents little difficulty. However, distinguishing potential instances of child abuse based on orofacial injuries can pose challenges [40]. The presence of multiple injuries and injuries at varying stages of healing should evoke suspicions of child abuse [41].

The inferential statistics regarding the distribution of etiology were found to be statistically significant (p=0.024). This indicates a clear association between the etiology of trauma and the specific type of injury sustained. Furthermore, a statistically significant correlation was observed between the specificity of the trauma and the type of injury sustained (p=0.002).

Polytrauma exhibited the highest prevalence (58.1%, n = 97), whereas 41.9% (n = 70) of the sampled cases presented isolated facial trauma. RTAs were the exclusive cause of all the polytrauma cases (100%, n = 97), highlighting the extensive trauma resulting from RTAs. On the other hand, only 35.7% (n = 25) of isolated facial trauma cases could be attributed to RTAs. Notably, most instances of isolated facial trauma were attributed to falls (40%, n = 28). This discrepancy demonstrated statistical significance (p=0.001).

Our study identified 167 individuals who experienced a combined total of 257 fractures. The upper third of the face exhibited the lowest frequency among these fractures. Specifically, frontal sinus fractures constituted nearly 4% (n = 10) of the entire fracture count.

Pediatric midface fractures comprised approximately 29% (n = 74) of the total fracture cases. This outcome aligns with a similar investigation conducted by Kenawy et al. [42]. The identification of mid-facial fractures among pediatric patients has become more frequent in recent times, likely influenced by the increased utilization of advanced imaging techniques. Notably, the study conducted by Van As et al. uncovered the inadequacy of conventional radiographs in accurately diagnosing mid-facial fractures [9,43]. Within the scope of our study, 74 (28.7%) fractures involving the middle third of the face were identified, with the majority classified as middle-energy zygomaticomaxillary complex (ZMC) fractures (10.1%, n = 26). Furthermore, in our study, zygomaticomaxillary complex (ZMC) fractures ranked as the most prevalent fractures in the middle third of the face (15.5%, n = 40). Correspondingly, Zhou et al. also determined ZMC fractures to be the second most common type when investigating the prevalence of maxillofacial trauma within pediatric North Chinese subpopulations [7]. Maxillary fractures involving Le Fort classifications contributed to nearly 6% (n = 15) of the total fracture cases. This finding contradicts the results from the study conducted by Mukhopadhyay et al., wherein maxillary fractures displayed a higher incidence than ZMC fractures [6].

Our study revealed orbital fractures constituting nearly 7% (n = 18) of all fractures. NOE fractures accounted for less than 1% (n = 1), which was less than the Zhou et al. study, where it was 4.2% [7]. Interestingly, they identified a lower incidence of orbital fractures than NOE fractures. Their research indicated that NOE fractures were twice as common as orbital fractures [7].

Fractures affecting the lower third of the face were the most prevalent in our study, comprising an incidence of 67.3% (n = 173) among all study participants. This notable prevalence aligns with findings observed across multiple studies [6,7,14]. Among these fractures, condyle fractures emerged as the most frequent, totaling 51 cases (19.8%). These results are consistent with the studies conducted by Thorén et al. and Segura-Palleres et al. [12,44]. Mandibular body fractures (n = 41) and parasymphysis fractures (n = 40) followed as the second most common type after condylar fractures. Each fracture type contributed to nearly 16% of the overall lower third of the face fracture count. Conversely, ramus fractures presented as the least encountered within the lower third of the face (1.1%, n = 3), and our study did not document any cases of coronoid process fractures.

Limitations of the study

The current study exhibits certain limitations, notably its retrospective nature. The primary challenge during this study was data acquisition, with numerous cases lacking proper documentation, such as treatment outcomes and the detailed nature of incidents. Another significant constraint stems from the exclusion of soft tissue and dentoalveolar trauma cases, given that data from ER visits that did not lead to admission were routinely expunged. In cases involving solely soft tissue injuries, such as lacerations, patients received initial management in the ER and were subsequently discharged for follow-up at the clinic. Conversely, dentoalveolar trauma prompted children to seek follow-up care with a pediatric dentist for splinting and vitality assessment. Additionally, it is crucial to acknowledge the potential for deliberate misreporting of the trauma's cause by individuals. This concern is particularly relevant in cases of interpersonal violence, where victims might attribute their trauma to a different factor out of fear or to downplay potential legal consequences [27]. To mitigate these limitations, a prospective randomized controlled trial is strongly recommended for future research endeavors.

## Conclusions

RTAs, trailed by incidents of falls, emerged as the primary instigator of maxillofacial traumatic fractures within a Saudi pediatric subpopulation treated at a tertiary trauma center. Fractures in the lower third of the face constituted more than two-thirds of the total fractures observed. Notably, condyle fractures exhibited the highest frequency among the observed fractures. Engaging in parental education regarding optimal child motor vehicle safety practices is advisable. Given that all instances of polytrauma were a consequence of RTAs, a thorough investigation into the specific causes of these accidents is warranted to curtail their incidence. Encouragement is extended towards conducting more comprehensive, multi-center studies to achieve a nationwide epidemiological and etiological assessment of pediatric maxillofacial trauma.
